# Application of explainable artificial intelligence integrating with electronic health record in oncology

**DOI:** 10.37349/etat.2026.1002357

**Published:** 2026-02-04

**Authors:** Yuhan Yang, Xici Liu

**Affiliations:** Agostino Gemelli University Policlinic, Italy; ^1^West China Hospital, Sichuan University, Chengdu 610000, Sichuan, China; ^2^Sichuan Technical Inspection Center for Medical Products (Sichuan Technical Inspection Center for Vaccine), Chengdu 610000, Sichuan, China

**Keywords:** explainable artificial intelligence, electronic health records, oncology, interpretability, model evaluation framework, SHAP, clinical decision support

## Abstract

Machine learning (ML) and deep learning (DL) models applied to electronic health records (EHRs) have substantial potential to improve oncology care across diagnosis, prognosis, treatment selection, and trial recruitment. However, opacity of many high-performing models limits clinician trust, regulatory acceptance, and safe deployment. Explainable artificial intelligence (XAI) methods aim to make model behavior understandable and actionable in clinical contexts. The present perspective summarizes current XAI approaches applied to EHR-based oncology tasks, identifies key challenges in evaluation, reproducibility, clinical utility, and equity, and proposes pragmatic recommendations and research directions to accelerate safe adoption in oncology. Common XAI categories used with EHR data include feature importance/interaction methods, intrinsically interpretable models, attention mechanisms, dimensionality reduction, and knowledge distillation or rule extraction. Tree-based models with SHapley Additive exPlanations (SHAP) explanations dominate recent EHR studies. Other interpretable strategies, such as generalized additive models and rule sets, appear in settings where transparency is prioritized. Gaps include inconsistent reporting, scarce formal evaluation of explanations for clinical utility, limited reproducibility for data and code availability, inadequate external validation, and insufficient consideration of fairness and equity that these issues are particularly important in oncology, where heterogeneity and stakes are high. Overall, integrating XAI with EHR-driven oncology models is promising but underdeveloped, which requires further progress by multi-stakeholder evaluation frameworks, reproducible pipelines, prospective and multicenter validations, and equity-aware design. The field should prioritize clinically meaningful explanations beyond ranking features and study how explanations affect clinician decision-making and patient outcomes.

## Introduction

Electronic health records (EHRs) provide rich, longitudinal, multimodal clinical data that can power machine learning (ML)/deep learning (DL) models, tasks including early detection, risk stratification, prognosis prediction, and treatment selection for oncology patients [1, 2], and optimizing clinical trial registration and enrollment for medical professionals [3]. High-performing “black box” models, such as ensembles and neural networks, frequently outperform traditional approaches on complex prediction tasks, yet their opacity impedes clinician trust, regulatory approval, and safe deployment in high-stakes oncology care [4–6]. Explainable artificial intelligence (XAI) techniques seek to open the black box by producing human-interpretable descriptions of model behavior and rationale [7–9]. Recent systematic and scoping reviews of XAI applied to EHR data emphasize a surge in XAI use but highlight notable heterogeneity and a lack of rigorous evaluation and reproducibility [10–12]. This perspective aims to synthesize those insights and apply them specifically to oncology, as well as outlining current practices, limitations, and actionable recommendations.

## XAI categorization relevant to EHR-based oncology

**Figure 1:**
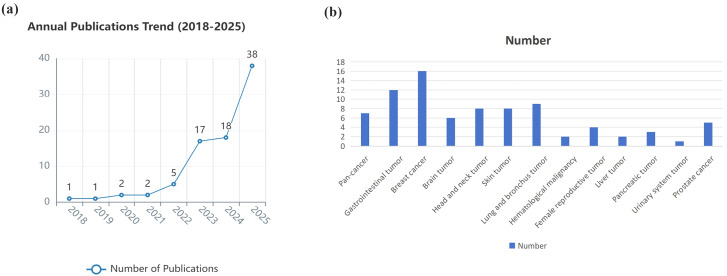
**Publication summary of XAI approaches used with EHR data in oncology.** (**a**) Publication trend of XAI studies in the field of oncology using EHR data between January 1st, 2018, and Nov 1st, 2025. (**b**) Oncology categorization of XAI studies using EHR data in recent publications. Panel (**a**) shows the number of publications per year meeting the inclusion; panel (**b**) categorizes included studies by oncology subdomain and XAI method. We searched PubMed, Web of Science, and arXiv for articles published Jan 1, 2018, to Nov 1, 2025, using terms (“explainable AI” OR “XAI”) AND (“electronic health records” OR “EHR”) AND (“oncology” OR “cancer”). Included studies applied XAI methods to EHR-derived oncology tasks. Exclusions: non-EHR imaging-only studies, purely technical XAI methods without EHR use.

Building on prior taxonomies, XAI approaches used with EHR data can be organized as follows. Each has different implications for clinical adoption in oncology. The publication trend related to XAI approaches in the field of oncology using EHR data in recent years is shown in Figure 1.

### Feature importance and interaction methods

The post-hoc, model-agnostic, or model-specific methods have been found to quantify the contribution of input features to model predictions, for instance, SHapley Additive exPlanations (SHAP), permutation importance, and partial dependence plots (PDPs) [13, 14]. Recent studies have highlighted SHAP’s widespread use in survival or stage prediction using ensemble/tree-based models on cancer cohorts, such as The Cancer Genome Atlas (TCGA) [15] that the application of SHAP could produce global feature rankings in which genes, mutations, or clinical covariates are most predictive of survival and local SHAP values to explain an individual patient’s risk. These kinds of XAIs have the potential to identify prognostic features like lab values, genomic markers, and comorbidities, explain individual risk predictions, and surface potential feature interactions, such as drug-comorbidity interactions.

### Intrinsically interpretable models

The models designed for transparency consist of generalized additive models (GAMs), decision trees, rule lists, and sparse linear models, which provide inherently understandable decision logic but may trade off predictive performance. The approaches like decision trees, logistic regression variants, and cluster‑based methods have been used for cancer diagnosis and risk prediction with interpretable survival scores from nomograms construction [16, 17]. The boosted/ensemble decision tree adaptations and microarray datasets have also been applied to produce interpretable models for cancer staging or subtype classification [18, 19]. It notes that the model development could be improved from an interpretable design, such as shallow decision trees, logistic regression with limited features, and Bayesian rule lists, rather than explaining a black box post-hoc. The optimized interpretability of XAI on EHRs would be used in treatment-decision aids with a clear rationale to generate triage tools guiding therapy escalation, as well as regulatory scenarios with required traceability.

### Attention mechanisms and representation-focused explanations

The sequence/time-series models have usually been used for longitudinal EHR data to highlight temporally important events and attention weights are sometimes interpreted as importance scores, although their reliability as explanations is debated. For clinical sequences, Reverse Time Attention model (RETAIN)-style reverse attention designed for interpretability and self-attention variants have been adapted for clinical sequences, that RETAIN/RetainVis-inspired models were applied to EHRs [20], which might have transferable potential to oncology longitudinal HER. Attention highlights prior imaging diagnoses, progressive lab trends, or particular chemotherapy cycles driving a prediction of near‑term deterioration or recurrence [15, 21, 22], which implies potential application on oncology treatment timelines to sequential HER by addressing critical time windows or events of rapid biomarker decline, driving a prognosis prediction.

### Dimensionality reduction and visualization

The methods, including t-SNE, UMAP, and concept bottlenecks, project high-dimensional features into lower-dimensional interpretable representations, which are useful for cancer cohort stratification and exploratory analyses, especially sparse DL, Least Absolute Shrinkage and Selection Operator (LASSO), clustering to identify exemplar patients, selecting gene pathways or features most informative for cancer subtype or survival [23–25]. The applied sparse DL and pathway-level selection have been applied in glioblastoma survival prediction using TCGA-like data, while dimensionality reduction has also found for gene and pathway discovery in cancer datasets [26]. The clustering or affiliation analysis on EHRs shows abilities in stratifying patients with stage 1 lung cancer for further definition of clinically meaningful subgroups [27].

### Knowledge distillation and rule extraction

There have been techniques for distilling complex model behavior into simpler surrogate models or rule sets to approximate decision boundaries, including surrogate decision trees, decision‑sets, rule extraction, and mimic learning. The mimic learning and distillation for clinical re-admission and mortality tasks have been discussed with applicable approaches to oncology models by distilling a neural survival model into a set of rules that estimate recurrence risk based on a small set of features [28]. The decision-sets design using metrics like rule count and rule length can be used to produce compact rule lists in order to classify tumor subtype or indicate eligibility for a clinical pathway [29].

## SHAP: benefits, known limitations, and recommended mitigations for oncology EHRs

SHAP has been widely used for feature-level explanations because of its theoretically grounded attribution and local explanations. However, several practical issues are critical in oncology applications:

- Instability and sampling variability: For complex, high-dimensional EHRs or rare endpoints (common in oncology), SHAP values can vary substantially with bootstrap resampling or small data shifts. Recommendation: report confidence intervals/variance for local SHAP attributions obtained via bootstrapping and quantify explanation reproducibility across folds.
- Feature correlation and attribution ambiguity: Correlated clinical or genomic features can produce misleading attributions since Shapley values distribute shared contribution among correlated features arbitrarily [30]. Recommendation: pre-process by grouping highly correlated features (e.g., pathway-level aggregation for genomics, summary lab trends), use conditional SHAP approximations when feasible, and complement with interaction analyses (SHAP interaction values) and causal or counterfactual checks.
- Computational burden: Exact SHAP for complex models and large feature sets is expensive. Recommendation: use approximations (TreeSHAP for tree ensembles), feature grouping, or mimic/surrogate models for explanations at scale; report compute resources and latency relevant for EHR integration.
- Interpretability in temporality/multimodality: Standard SHAP applied to tabular snapshots may miss sequential or imaging features’ contributions. Recommendation: apply SHAP to modality-specific components (e.g., per-visit features, embedding dimensions for images) and combine with temporal attention visualizations and counterfactual narratives.

## Applications and further considerations of XAI integrating EHR

For the diagnosis of oncology patients, the EHR-based predictors for early cancer detection benefit from explanations in flagging at-risk patients from routine labs and symptoms by justifying screening or referral recommendations and avoiding alarm fatigue [31, 32]. Explaining survival or recurrence risk allows oncologists to contextualize model outputs with patient-specific drivers on tumor markers and performance status, which enables shared decision-making for prognosis and survival prediction [33]. For patients who need precise treatment, XAI reveals clues about which clinical, genomic, or comorbidity features could drive predictions favoring a given therapy, potentially supporting treatment personalization by offering interpretability when model recommendations diverge from standard of care [34]. To optimize clinical trial matching and operational workflows, transparent models can justify eligibility flags and improve trial recruitment by allowing trial teams to understand why patients are prioritized [35, 36]. Otherwise, explanations could strengthen treatment selection by helping clinicians assess whether a predicted adverse event is plausible and what modifiable factors contributed to safety monitoring [37].

It has been found a lack of rigorous evaluation of XAI methods for clinical relevance that typical assessments often stop at qualitative plausibility or visualization [38]. For further oncology deployment, it requires comprehensive evaluations by addressing fidelity about how well explanations reflect true model computations, clinical usefulness of whether explanations change clinician decisions appropriately, robustness in stability of explanations across input perturbations and cohorts, and human-centered evaluation concerning usability studies with oncologists and multidisciplinary teams. Otherwise, reproducibility remains limited that many studies have used proprietary EHRs without sharing code, independent verification, or external validation [33, 39, 40]. The XAI models in area of oncology must prioritize data-sharing strategies, code release, and detailed reporting of preprocessing and model pipelines. Moreover, clinician trust depends not only on explanation correctness but also on relevance, presentation, and fit within clinical workflows. Therefore, explainability should be tailored that simple feature lists might suffice for some tasks with binary classes while causal or counterfactual explanations might be more persuasive for treatment decisions. To identify legal and regulatory factors, regulatory frameworks increase the imperative for meaningful explanations. For oncology patients with life-altering interventions, regulators and institutions will expect rigorous validation and traceability.

## Challenges and future directions of XAI in oncology

There have been several challenges influencing future application of XAI on EHRs in oncology, including heterogeneity, multimodal data, class imbalance, and equity and representation. Heterogeneity is attributed to tumor biology diversity, treatment modalities, and patient comorbidities, which increase model complexity, as well as the challenge of producing generalizable explanations. For multimodal data, oncology relies on imaging, pathology, genomics, and EHR-derived clinical features that integrating and explaining multimodal models remains technically and conceptually difficult. Otherwise, there exists class imbalance. Many oncology endpoints are rare, which make model explanations sensitive to sampling variability. Concerning healthcare equity, under-representation of low-resource settings and minority populations risks biased models and explanations without generalization, which would be raised as a major equity gap in AI-in-healthcare reviews and domain-specific syntheses.

There still be several oncology-specific challenges and implications:

- Heterogeneity: inter-tumor and intra-patient heterogeneity mean features predictive in one cohort may not generalize. Action: require external validation and subgroup explanation audits; present explanation differences by tumor subtype/stage.
- Sparsity and rare events: many oncology endpoints (e.g., rare toxicities) lead to class imbalance, making explanation reliability low. Action: bootstrap estimation of explanation variance; use data augmentation or synthetic controls to stabilize estimates.
- Multimodality: EHRs combined with imaging, pathology, and genomics complicate explanation attributions. Action: use modality-specific explainers, hierarchical explanations (first indicate which modality, then which features), and design visualization for tumor boards.
- Temporal complexity: treatments and biomarker trajectories matter. Action: adopt sequence-aware explainers (RETAIN-like models with clear attention maps, counterfactual time-window analyses) and present temporal narratives.
- Equity/regulatory stakes: life-altering decisions require rigorous fairness auditing and traceability. Action: add fairness metrics and transparent documentation of datasets and preprocessing.

The further application of XAI on EHRs in oncology needs to prioritizing clinically meaningful explanation types by moving beyond feature importance ranks to context-rich explanations, for example, counterfactuals about what would change the prediction, causal insights where feasible, and patient-level narratives connecting model drivers to actionable clinical considerations. The standardized evaluation framework is also warranted by adopting batteries to assess fidelity, robustness, clinical utility, and user acceptance and incorporating prospective human-in-the-loop studies to measure decision changes and patient-level outcomes. The enhancement of reproducibility and reporting is also required by sharing code, model checkpoints, synthetic or de-identified datasets when possible as well as adhering to reporting checklists including data preprocessing, feature construction, model selection, and XAI settings. Otherwise, the requirement of external validation across diverse oncology centers with prospective pilot deployments need to be implemented to assess transportability and real-world impact. With equity-centered design and fairness auditing, systematical evaluation of subgroup performance and explanation differences consists of fairness metrics and qualitative assessments with clinicians caring for under-represented populations. Furthermore, it’s necessary to develop interactive explanation interfaces integrated into oncology workflows, such as EHR dashboards and tumor boards co-designed with clinicians to present explanations at the appropriate level of detail.

## Multi-dimensional evaluation and integration framework for XAI in oncology EHRs

A practical framework has been proposed to evaluate and integrate XAI into oncology workflows. The framework explicitly evaluates explanations across five dimensions and prescribes integration steps:

- Fidelity: quantitative checks that explanations reflect model computation (e.g., agreement between surrogate and original model, perturbation tests).
- Robustness and stability: sensitivity analyses across input perturbations, bootstrapping to measure explanation variance, and tests across cohorts [41].
- Clinical utility: prospective human-in-the-loop studies assessing whether explanations change clinician decisions in intended directions; pre/post comparison of decision concordance and downstream patient-relevant outcomes.
- Fairness and transportability: subgroup performance and explanation disparity audits across demographic, socioeconomic, and clinical strata; external validation across centers.
- Usability and workflow fit: qualitative usability testing with oncologists, nurses, and tumor board members, integrated User experience (UX) for EHR dashboards, and contextualized explanation level (summary vs. detail).

### Integration steps

- Pre-deployment: internal fidelity and stability tests + fairness audits on retrospective data.
- Pilot deployment: prospective small-scale human-in-the-loop pilot within tumor boards; measure decision changes and feasibility.
- Multicenter validation: external validation at heterogeneous centers, with standardized reporting and code release.
- Deployment & monitoring: continuous post-deployment monitoring of model and explanation drift and periodic re-audits.

### Use-case: explainable multimodal model for recurrence risk in stage II–III colorectal cancer

- Dataset: de-identified EHR (demographics, labs, medications, comorbidities), pathology reports (NLP-extracted features), targeted genomic panel (mutations), and structured treatment records (retrospective).
- Task: 2-year recurrence risk prediction after definitive surgery (binary), with class imbalance.
- Model architecture: multimodal fusion model using gradient boosted trees (LightGBM) on tabular EHR plus logistic regression on aggregated genomic pathway scores; output fused via stacking ensemble to predict recurrence probability. Temporal features encoded as summary trends (slope, variance) over pre-surgery period.
- XAI pipeline: TreeSHAP for global and local attribution on ensemble predictions; bootstrap SHAP variance estimates; grouped features for genomic pathways and lab trends; counterfactual generation (sparse counterfactuals) for actionable narratives (“If neutrophil-to-lymphocyte ratio had been X lower, predicted risk would fall by Y%”).
- Evaluation: (1) Fidelity: compare surrogate decision tree accuracy with original model (ΔAUC), (2) Robustness: bootstrapped SHAP confidence intervals and perturbation tests, (3) Clinical utility: within a tumor board pilot, randomized case vignettes with vs. without explanations to measure change in treatment recommendations and confidence, (4) Fairness: subgroup AUC & explanation disparity by race/insurance status, (5) Reproducibility: share code and synthetic dataset; provide preprocessing scripts.

## Conclusions

In conclusion, explainable AI methods hold promise to unlock EHR-driven advances in oncology by improving interpretability, trust, and the safe translation of predictive models into clinical practice. The current practices have been characterized by rapid uptake of post-hoc explanation tools notably SHAP but insufficient evaluation, reproducibility, and equity assessment. For application of XAI on EHRs in oncology, future work must emphasize rigorous evaluation of explanations, multicenter validation, transparency, and human-centered design to ensure the clinically meaningful, equitable, and trustworthy support delivered by XAI.

## Abbreviations

DL: deep learning
EHRs: electronic health records
PDPs: partial dependence plots
RETAIN: Reverse Time Attention model
SHAP: SHapley Additive exPlanations
TCGA: The Cancer Genome Atlas
XAI: explainable artificial intelligence

## Declarations

### Copyright

© The Author(s) 2026.
